# Inflammaging, an Imbalanced Immune Response That Needs to Be Restored for Cancer Prevention and Treatment in the Elderly

**DOI:** 10.3390/cells10102562

**Published:** 2021-09-28

**Authors:** Juana Serrano-López, Beatriz Martín-Antonio

**Affiliations:** Departmentof Experimental Hematology, Instituto de Investigación Sanitaria-Fundación Jiménez Diaz, IIS-FJD, Autonomous University of Madrid, 28040 Madrid, Spain; juana.serrano@quironsalud.es

**Keywords:** inflammaging, immunosenescence, SASP, immunotherapy, T cells, NK cells

## Abstract

Nowadays, new advances in society and health have brought an increased life expectancy. However, at the same time, aging comes with complications that impact the development of autoimmunity, neurodegenerative diseases and cancer. These complications affect the quality of life and impact the public health system. Specifically, with aging, a low-grade chronic sterile systemic inflammation with self-reactivity in the absence of acute infection occurs termed inflammaging. Inflammaging is related to an imbalanced immune response that can be either naturally acquired with aging or accelerated due to external triggers. Different molecules, metabolites and inflammatory forms of cell death are highly involved in these processes. Importantly, adoptive cellular immunotherapy is a modality of treatment for cancer patients that administers ex vivo expanded immune cells in the patient. The manipulation of these cells confers them enhanced proinflammatory properties. A general consequence of proinflammatory events is the development of autoimmune diseases and cancer. Herein, we review subsets of immune cells with a pertinent role in inflammaging, relevant proteins involved in these inflammatory events and external triggers that enhance and accelerate these processes. Moreover, we mention relevant preclinical studies that demonstrate associations of chronic inflammation with cancer development.

## 1. Introduction: Immunosenescence and Inflammation during Aging, and its Consequences in Cancer and Other Age-Related Diseases

Nowadays, the elderly population (>65-year-old) in Europe represents 19.7% of the population. This number is predicted to continue increasing and reach 28.5% in 2050 [1]. With that in mind, those numbers will impact social life and public healthcare. Thus, a new discipline termed “Geroscience” has emerged to decipher the link between mechanisms of aging and susceptibility to age-related diseases [2,3].

Biologically, aging is associated with a physiological process of tissue degeneration related to chronic inflammation [4]. This age-related chronic inflammation is highly associated with inflammaging, which was initially defined as a progressive increase of proinflammation in aged organisms [5], leading to increased morbidity and mortality [6]. Currently, inflammaging is defined as the elevated low-grade chronic sterile systemic inflammation with self-reactivity in the elderly in the absence of acute infection [7].

“Immunosenescence”, a process associated with aging that impairs the immune function, is highly responsible for inflammaging. Different age-associated events cause immunosenescence. Specifically, during aging, occurs a thymic involution that reduces the pool of naïve T cells and amplifies the oligo-clonal expansion of memory T cells. These events will cause a reduced immune repertoire diversity [8], leading to reduced ability to fight infections and increased cancer incidence [9]. Thymic involution also leads to an amplified release of self-reactive T cells and reduced capacity of T-regulatory (reg) cells to suppress these self-reactive T cells and preserve immune homeostasis. Consequently, these events will enhance tissue damage with autoimmunity and chronic inflammation, being essential contributors to inflammaging [7,10].

Immunosenescence also occurs in the BM, which constitutes the primary site of hematopoiesis [11]. Thus, aging causes both a gradual replacement of the different cellular components of the BM by adipocytes and a skew towards the generation of myeloid cells [12]. These changes negatively impact the repertoire and activity of T and B lymphocytes [13]. Moreover, this cellular degeneration in the BM will increase the production of proinflammatory cytokines [12,14,15], impacting the activity of immune cells.

The innate immune system is also impacted by immunosenescence. Thus, neutrophil and macrophage capacity for phagocytosis and subsequent elimination of dead cells is reduced with aging [16,17]. Macrophages also acquire an increased polarization towards M2 cells [18], and natural killer (NK) cells present a reduced capacity to secrete cytotoxic molecules [19].

Overall, immunosenescent cells will not be able to remove senescent somatic cells that also accumulate with aging [20,21,22,23,24] and are characterized by secretion of proinflammatory molecules known as senescence-associated secretory phenotype (SASP) [25]. The SASP is another crucial contributor to inflammaging [7,25]. This accumulation of senescent cells will enhance the SASP promoting further inflammaging and accelerated aging [22] and will contribute to cancer development [7,25,26]. Furthermore, the SASP transmits cellular senescence to neighboring non-senescent cells [27,28], leading to enhanced senescence and inflammaging.The SASP is also increased with anti-cancer therapies that induce senescence in both immune and tumor cells, leading to enhanced inflammation and treatment resistance [29,30].

Moreover, microbes debris of exogenous origin and cell debris of endogenous origin are recognized through the pathogen-associated molecular pattern (PAMPs) and damage-associated molecular patterns (DAMPs), respectively [31], the latter being part of the SASP [32]. PAMPs and DAMPs become more abundant during aging, and PAMP stimulation induces DAMP secretion by immune cells [33], leading to enhanced inflammaging.

This feedback occurring between immunosenescence and inflammaging explains the involvement of both processes in age-related diseases, including cancer, neurodegenerative diseases, metabolic diseases and cardiovascular diseases [7] (see Table 1). For instance, Alzheimer’s disease is a chronic neurodegenerative disease with pathological accumulation of amyloid-beta (Aβ) peptides and neurofibrillary tangles containing tau protein. Aβ and tau deposition cause an age-dependent deterioration of the blood-brain barrier that leads to the infiltration of immune cells into the central nervous system exacerbating the neurodegenerative process and promoting inflammatory responses [33]. Type-2 diabetes is a multifactorial metabolic disease with chronic hyperglycemia and dyslipidemia as main pathological features. A chronic low-grade inflammation resembling inflammaging induces insulin resistance and dysfunction of β-cells, emerging as a relevant factor contributing to the development of diabetes [34].

In cancer, aging and chronic inflammation are highly involved in its development [35,36]. However, the intricate relationship between aging and cancer is not clear. In detail, half of the cancers occur in individuals older than 70. Yet, whereas aging and cancer share disease mechanisms, such as genomic instability, they also present opposite features, such as hypoactive cells in aging vs. hyperactive cells in cancer [36].The role of chronic inflammation in cancer is also controversial. Thus, inflammation is required initially for immune surveillance; however, failure to resolve inflammation will promote tumor growth [37,38]. The relevant impact of chronic inflammation in cancer is suggested by different studies that estimate that 15–20% of cancers are inflammation-related [39]. 

For instance, autoimmune diseases such asinflammatory bowel disease (IBD) increase the risk of developing colorectal cancer [47]. Moreover, numerous studies have revealed associations of high levels of inflammatory markers, such as C-reactive protein (CRP) [44] and IL6 [45], with an increased risk of developing different types of cancer.

The relevant role of chronic inflammation in cancer and of the immune response in the development of inflammaging should be considered incancer patients treated with adoptive cellular immunotherapy. These treatments administer various immune cells in patients, such as chimeric antigen receptor (CAR)-T cells [48], tumor-infiltrating lymphocyte (TIL) [49] or NK cells [50] which previously have been modified and expanded in vitro. The in vitro expansion changes the phenotype of immune cells and their cytotoxic mechanisms that activate inflammatory forms of cell death [50,51,52,53]. For instance, after encountering tumor cells, CAR-T cells [53] and NK cells [44] initiate pyroptosis, an inflammatory form of cell death. Pyroptosis was initially described as a type of cell death triggered by the innate immune system after recognition of intracellular pathogens by intracellular receptors. Nucleotide-binding oligomerization domain (NOD)-like receptors (NLRs), and among them NLRP3, belong to these receptors. They initiate the assembly of inflammasomes that will activate caspase-1 leading to release of IL1β and IL18 and the pore-forming protein gasdermin-D (GSDMD) the latter inducing pyroptosis [54]. Of interest, in CAR-T cellimmunotherapy, NLRP3 activates pyroptosis with release of DAMPs, IL1β and IL6 [53]. In addition, immune cells after encountering tumor cells release different types of Granzymes (Gzm). Besides the classic GzmB, other inflammatory Gzm, such as GzmA and GzmK, are involved in the anti-tumor activity of immune cells [55,56].

These relevant associations of inflammaging with an inadequate immune response and the development of inflammatory diseases and cancer, added to the fact that cancer associates with aging suggest their relevance in the field of cellular immunotherapy. Here, we will review the contribution to inflammaging of different subsets of T cells and NK cells, as they are administered in cancer patients, either unmodified or modified with a CAR [51,57,58]. Moreover, the role of NLRP3 and inflammatory granzymes, activated during the innate and adaptive immune response, will be presented, focusing on their impact on inflammation. Other intrinsic and external inflammation triggers related to cancer will be mentioned, and some preclinical models that associate inflammation with cancer development will be cited.

## 2. Variation of T-reg Cells and Th17 Cells during Aging and Their Impact on the Development of Inflammaging

Human centenarians represent a model with low inflammaging to study healthy aging. Of interest, although those human centenarians present a systemic inflammatory state (e.g., high levels of IL6 and IL8 in plasma), they also count on efficient anti-inflammatory networks termed anti-inflammaging that compensate for inflammaging [59]. Analyses of the immune cell populations in centenarians have concluded that longer survivors present higher leucocytes with a higher number of naïve, activated/memory and effector/memory CD4 and CD8 T cells [60]. Proteomic studies in centenarians also show a pattern with less inflammaging and autoimmunity, increased B cell-mediated immune response, higher expression of proteins involved in angiogenesis and enhanced intercellular junctions [61].On the other side, elderly cancer patients, such as multiple myeloma (MM), present immunosenescent T cells with deficient immune responses [62] that will increase inflammaging.

Among the different subsets of T lymphocytes, we will focus on T-reg and Th17 cells that share a common precursor and present opposing roles in developing inflammaging. Thus, Th17 cells cause autoimmunity and inflammation, and T-reg cells inhibit their activity [63]. Specifically, during aging, there is an increased production of Th17 cells that will contribute to inflammaging [64] and a decrease in the functionality of T-reg cells that will increase chronic inflammation [65]. Even though Th17 cells are very well-known for their role in inflammation and autoimmunity, their role in cancer is less understood. Notably, an intricate balance between T-regs and Th17 cells must be maintained to avoid developing these pathologies [64,66,67].

### 2.1. Changes in the T-reg Cell Compartment during Aging and Impact in Inflammation and Cancer

The impact of T-reg cells during aging should be analyzed considering the variation in numbers and their functionality. As previously mentioned, thymic involution with aging reduces the capacity of T-reg cells to suppress self-reactive T cells and preserve immune homeostasis [7,10]. Two different origins have been described for T-reg cells. The first one is the thymus, at the early stages of life, which gives rise to naturally occurring T-reg (nT-reg) cells after escaping from the negative selection in the thymus, followed by appropriate TCR stimulation. The second one is in the peripheral blood (PB) and secondary lymphoid organs, where different triggers induce the expression of Foxp3 in naïve T cells, originating inducible T-reg (iT-reg) cells. iT-reg cells have a similar phenotype and suppressive function to nT-regs [68]. Data suggest that aging induces a decline in iT-regs and an increase in the number of nT-reg cells [69].

Regarding the functionality, it remains controversial whether aging induces a loss of T-reg functionality or just an effect of the variation in the number of T-reg cells [69]. Studies in aged mice have observed an increased proportion of functional CD4 T-regs in PB and lymphoid tissues, decreasing the effector T cell responses against *Leishmania* infection [70]. In humans, there is also an increase in the number of CD4 T-regs in PB with immunosuppressive properties [71]. In addition, the increased number of T-reg cells with aging can be explained by the polarization of CD4 conventional T cells to cells with T-reg cell properties, an event observed in aged mice [72]. Moreover, CD8 T-reg cells are a relevant immunosuppressive cell population [73], increasing with aging in absolute numbers in PB, the spleen and lymph nodes presenting functionality [74,75].

Various studies have associated the functionality of T-regs with the progression of different tumors due to their immunosuppressive activity [76,77]. Thus, in melanoma and colon carcinoma models, intratumoral T-reg cells inhibit the anti-tumor activity of TILs [78]. In these models, T-reg cells can adapt to the lactic acid-enriched TME through CD36 up-regulation that enhances their mitochondrial fitness [78]. In MM, where most patients represent an elderly cancer population, elevated frequencies of functional T-reg cells are present in newly diagnosed and relapsed patients compared to healthy volunteers [79].

On the other side, autoimmunity and chronic low-grade inflammation, both hallmarks of inflammaging [80], are also recognized as drivers of cancer [35,39]. In this scenario, murine models of autoimmunity have shown the beneficial impact of T-reg cells ameliorating inflammation. For instance, in models of multiple sclerosis, T-reg cells produce CCL1 that upregulates its receptor, CCR8, and induces the expression of CD39, granzyme B and IL10, which suppress the disease [81]. In autoimmune colitis, aged T-reg cells present equal suppressive in vitro activity than young T-regs to mitigate the disease. In these models, aged T-reg cells were able to restrain IFN-γ T cell responses. Even though, they controlled Th17 cells only in cases of acute inflammation and not in cases of chronic inflammation, leading to autoimmunity and promoting colitis [82]. T-reg cells also present contradictory roles in inflammation. Thus, IBD is another autoimmune disease that increases the risk of developing colorectal cancer [47]. In this scenario, different murine models have demonstrated the protective role of T-reg cells in IBD development through the suppression of T effector cells. In detail, IL35 secretion by T-reg cells suppresses the proliferation of effector T cells. However, on the other side, IL35 overexpression associates with the induction of gastrointestinal cancer [83,84].

Another relevant model that contradicts the relationship of chronic inflammation mediated by T lymphocytes and cancer and where T-reg cells are involved is the graft versus host disease (GVHD). Chronic GVHD (cGVHD) is a relevant complication after allogeneic stem cell transplantation (allo-SCT) mediated by donor’s T lymphocytes that enhances mortality due to a chronic inflammatory response and at the same time reduces the risk of cancer relapse [85]. T-reg cells associate with reduced development of GVHD [86]. Of interest, pediatric allo-SCT recipients have a lower incidence of cGvHD than adults [87], which might reflect in this context the beneficial impact of lower immunosenescence levels in pediatric patients compared to adult patients. Indeed, it has been observed that cGVHD-derived T-cells present high expression of genes that positively regulate cellular senescence (*CDKN2A*, *SERPINB9*, *LYPLA1* and *CKTM1A/B*) [88].

To summarize, two opposite scenarios,"enhanced immunosuppression and chronic inflammation", associate with cancer, and T-reg cells play either a detrimental or beneficial role in both systems. These findings bring the question of the exact contribution of T-reg cells in the regulation of inflammation and cancer development, specifically in the elderly.

### 2.2. Th17 Compartment and Its Delicate Balance with T-reg Cells

Th17 cells are critical players in maintaining mucosal immune homeostasis and protection against pathogens. They are also very well-known for their role in inflammation and autoimmunity. An intricate balance between T-regs and Th17 cells is maintained to avoid developing these pathologies [66,67]. A common precursor for T-reg cells and Th17 cells will differentiate into one cell subtype depending on the cytokine environment [67]. In detail, TGFβ is required for differentiation from naïve CD4 T cells to both Th17 and iTreg. Thus, TGFβ upregulates the retinoic acid-related orphan receptors-γt (RORγt) and Foxp3, which give rise to a common precursor of T-regs and Th17 cells. In the presence of TGF-β, both IL6 and IL21 induce differentiation to Th17 cells. Otherwise, T cells will differentiate to T-reg cells. Moreover, Foxp3 inhibits Th17 development through binding to RORγt. Without IL6, TGFβ reinforces this inhibition and favors the formation of T-reg cells. In addition, Th17 and T-reg cells can also polarize to each other [67].

The role of T-reg cells in maintaining the number of Th17 cells has been observed in different contexts. For instance, intestinal T-reg cells constrain microbiota-dependent IL-17-production by Th17 cells. This activity is dependent on the transcription factor c-Maf that controls IL10 production by T-reg cells [89]. In a murine model of neuroinflammation, imaging of T-reg and Th17 cells in the spinal cord demonstrated that T-regs suppress Th17 cells by inhibiting Ca^2+^ signaling and limiting the access of Th17 cells to APCs, avoiding neuroinflammation [90]. On the contrary, in hepatic carcinoma, increased Th17 levels are detected in the PB, correlating positively with metastasis progression and T-reg cells in the TME [91].

Altogether, T-reg and Th17 cells present opposite roles with an intricate regulation between them. Monitoring their changes in elderly cancer patients and patients receiving adoptive cellular immunotherapy will provide relevant information in this field.

### 2.3. Changes in the Th17 Compartment during Aging and Implications for Autoimmunity and Cancer

Aging causes an increased Th17/T-reg ratio that contributes to inflammaging [92]. Indeed, older subjects present higher Th17 cytokine production than younger subjects. One of the causes described is defective autophagy in CD4 T cells occurring with aging, leading to reduced mitophagy with an accumulation of malfunctioning mitochondria. These events result in the upregulation of Th17 cytokines contributing to inflammaging [64,93]. The detrimental impact of this higher Th17/T-reg ratio in cancer is observed at specific stages of tumors. Thus, oral squamous cell carcinoma patients increase the Th17/T-reg ratio at early stages and decrease it at late stages [94]. In colorectal tumor specimens, patients with increased expression of Th17 genes presented a poor prognosis [95]. Others have found that the increased IL1β and IL2 reduction in aged mice contributed to an elevated Th17 differentiation [96].

In MM, a variety of studies confirm the detrimental role of Th17 cells. Thus, Th17 cells promote MM growth and inhibit immune functions [97]; and in MM patients with lytic bone disease, numbers of Th17 cells were the highest [98]. Of interest, IL6, which is over-expressed in MM, creates a proinflammatory TME, a crucial factor mediating the conversion of T-regs into Th17 cells [99]. Th17 cells also cause osteoclast-dependent bone damage in vitro and in vivo, where miR-21 activates differentiation of naïve T cells in Th17 cells, promoting these detrimental effects in MM [100]. IL17, produced by Th17, cells induces osteoblasts pyroptosis in vitro, through activation of the NLRP3 inflammasome complex with Caspase-1 execution and release of IL1β [101]. In newly diagnosed MM patients, Th17 cell levels fluctuate considerably. Of interest, Th17 increased further when the disease reached partial remission, decreased to normal levels when complete remission was achieved and increased again when the disease recurred [102].

Moreover, in MM, dendritic cells (DCs) infiltrate the BM as efficient inducers of Th17 cells and promote higher levels of Th17 in BM than PB. Of interest, in monoclonal gammopathy of undetermined significance (MGUS) patients, an initial stage of the MM disease, this increase in Th17 cells was not observed. Another study analyzing the microbiota in MM observed that *Prevotella heparinolytica* promotes the differentiation of Th17 cells that colonize the gut and migrate to the BM, to favor the progression of MM. Similarly, in smoldering MM patients, higher BM IL17 levels predicted faster disease progression [103].

Moreover, the imbalance of the Th17/T-reg ratio in MM is reinforced by studies, where MM and MGUS patients show a reduction in the number of T-reg cells compared to healthy donors, being these T-reg cells dysfunctional [104]. Another study observed fewer T-regs in the BM of MM patients compared to healthy individuals, where Th17 cells are responsible for osteoclast activation mediating lytic bone disease [105].

To summarize, Th17 cells are highly involved in this connection between chronic inflammation and cancer development. Moreover, they are related to different types of cancer and to the pathogenic events of MM patients, who represent elderly cancer patients. Novel studies are required to decipher their role in the progression of these diseases. 

## 3. Variation of NK Cells during Aging and Cancer and Their Impact on the Development of Inflammation

### 3.1. Immunoregulatory Activity of CD56^bright^ NK Cells

NK cells classify into two big groups, the mature and cytotoxic CD56^dim^ NK cells, constituting 90% of PB NK cells, and the immature and immunoregulatory CD56^bright^ NK cells form 10% of PB NK cells [51]. NK cells present an important anti-tumor, immunoregulatory and antimicrobial activity that has made them an attractive target in cancer and autoimmune diseases [51,56,106,107]. However, there are conflicting results about the protective or pathogenic role of NK cells in autoimmunity; and in cancer, their anti-tumor activity is far from the action reached by CAR-T cells [51].

In the context of inflammation and viral infection in healthy individuals, CD56^bright^ NK cells suppress autologous CD4 T cell proliferation through direct cytotoxicity, dampening the inflammatory process. Thus, they inhibit autologous CD4 T cell proliferation in autoimmunity [108]; and in viral infections to avoid an excessive inflammatory response that might be lethal to the host [109]. However, in pathological conditions, such as multiple sclerosis, they display a lower ability to do it, which is related to increased HLA-E expression on T cells, the ligand of the inhibitory NKG2A receptor on NK cells [108]. This beneficial role of CD56^bright^ NK cells avoiding inflammation is confirmed when multiple sclerosis patients that respond to treatment and present remission of the disease during pregnancy show an expansion of CD56^bright^ NK cells. Of interest, there is increased relapse after the pregnancy with loss of CD56^brigh^t NK cells [110]. In this regard, during pregnancy, a subtype of CD56^bright^ NK cells increases, known as decidual NK cells [111], which might explain this association observed [110].

This inhibition of autologous CD4 T cell proliferation by NK cells can be mediated through the immunosuppressive molecule adenosine, which is defective in juvenile idiopathic arthritis patients. Lower NK cell activity could be related to a different expression of adenosine receptors in these patients and reduced CD38/CD73 expression [112]. Inhibition of CD8 T cells by NK cells has also been observed in murine models of type I diabetes through the expression of CD117 and PD-L1 on NK cells that limit the disease’s development [113].

### 3.2. Variation of NK Cells in Aging and Cancer

With healthy aging, a decrease in the CD56^bright^ NK cell subset and an increase in the CD56^dim^ subset is observed, which might be related to a lower ability to control inflammatory responses with aging [114]. Moreover, healthy individuals older than 85 presented NK cells with higher expression of SIRT1 and HSP70, proteins related to response to stress, suggesting that the most aged healthy seniors develop an increased NK cell response to adaptive stress [115].

In elderly cancer patients, for instance, MM, there are more NK cells than in healthy donors, and as in healthy elderly, there is an increase in the CD56^dim^ NK cell population with low expression of CD94, which defines the progression of CD56^bright^ towards CD56^dim^ NK cells. Moreover, this subset of NK cells eventually accumulates at the different stages of the disease progression (MGUS, smoldering MM and MM) [116].

The role of CD56^bright^ and CD56^dim^ NK cells in the progression of cancer patients follows distinct patterns depending on the disease. Thus, in acute myeloid leukemia (AML), which also represents a disease with increased incidence in the elderly [117], a beneficial impact for CD56^bright^ NK cells is not observed. On the contrary, AML patients could be grouped into three distinct groups according to the maturation stage of NK cells, where hypomature NK cells showed the worst prognosis. This hypomaturation state was associated with a reduced frequency of memory-like NK cells [118]. The activating NK receptors NKp30, NKp46 and DNAM-1, mediating NK anti-tumor activity, are decreased in the elderly and AML patients [114]. Of interest, AML blasts are involved in the loss of NKp30 and NKp46 in AML patients, a process that is reversed when achieving complete remission [119,120]. Another study in AML patients showed that NK cells in BM presented stress-induced repression of NK cell effector functions and reduced CD160 levels correlated with lower survival [121]. Moreover, an accumulation of CD56^−^CD16^+^ unconventional NK cells, which correlates with poor prognosis, is observed in AML. These NK cells had decreased NKG2A, NKp30, NKp46, NKG2D, DNAM-1 and CD96 [122]. Other contradictory results are observed. For instance, in bladder cancer, NK cells are the predominant intratumoral lymphocytes, where CD56^bright^ NK cells associate with improved survival and CD56^dim^ NK cells with higher pathological stage [123]. In advanced melanoma patients, abundance of CD56^bright^NK cells is detected in PB that correlates negatively with survival and distant metastases [124].

These contradictory results about the beneficial and detrimental role of CD56^bright^ NK cells suggest an intricate balance that needs to be maintained for the different NK cell subsets during the immune response. Indeed, immunoregulatory CD56^bright^ NK cells, could be compared to decidual NK cells, which are immune-tolerant and characterized by CD56^bright^CD16^−^CD9^+^CD49a^+^ and Eomes^+^ expression [125,126]. Importantly, they produce large amounts of proangiogenic factors, including VEGF, PlGF, CXCL8, IL10 and angiogenin [127], that might contribute to their detrimental associations observed in some cancer studies. Of interest, NK cells administered in immunotherapy treatments become CD56^bright^ after the in vitro expansion [50]. Therefore, analysis of the variation of NK cell phenotypes during immunotherapy studies will provide relevant information.

## 4. Impact of NLRP3 and Granzymes in Pyroptosis during the Immune Response and the Development of Inflammation

As mentioned in the introduction, cells of the innate immune system have an essential role in developing inflammaging. Specifically, the innate immune response recognizes PAMPs and DAMPs to become activated. This recognition is performed through pattern-recognition receptors such as toll-like receptors (TLRs) and NLRs that are part of the inflammasome. The inflammasome cleaves proinflammatory cytokines into a mature form that will alert the immune system of potential dangers initiating a proinflammatory reaction [31,128]. Of interest, immune cells administered in immunotherapy also activate the NLRP3 inflammasome that triggers pyroptosis, enhancing inflammatory responses [52,53]. In addition, once immune cells become activated, they will release inflammatory Gzm [55,56] that will impact not only the anti-tumor activity but also the development of inflammation. Here, we summarize the impact of NLRP3 and inflammatory Gzm in developing inflammatory diseases and the anti-tumor efficacy of immune cells.

### 4.1. Impact of NLRP3 in Inflammatory Diseases

NLRP3 is the best-studied NLR protein of the inflammasomes, implicated in different pathologies related to inflammation [129,130,131]. NLRP3 activates caspase-1 thatcleaves pro-IL1β and pro-IL18 into their mature forms. Activated caspase-1 also cleaves GSDMD that will form lytic pores in the cell to promote pyroptosis, facilitating the release of mature IL1β and IL18. IL1β and IL18 will bind to receptors on other cells to initiate and propagate this inflammatory response to clear the threat [132]. Unfortunately, the release of IL1β and IL18 will also impact the development of inflammatory diseases. Thus, the aberrant release of IL1β is involved in the pathogenesis of a range of inflammatory diseases, such as gout, type II diabetes, atherosclerosis, obesity, heart failure, recurrent pericarditis, rheumatoid arthritis and smoldering myeloma [40]. For instance, in type II diabetes, the aberrant activity of the NLRP3-inflammasome complex leads to IL1β secretion and caspase-1 activation that impair pancreatic β-cell function, adipocyte function and insulin sensitivity, promoting obesity and insulin resistance [42,43]. In atherosclerosis, cholesterol crystals activate NLRP3 in macrophages inducing acute inflammation [133], and inhibition of NLRP3 ameliorates atherosclerosis progression [134,135]. Of interest, these diseases are responsive to IL1β neutralization [40].

NLRP3 contributes to the inflammatory activity of Th17 cells and their negative impact on inflammatory diseases. Specifically, rheumatoid arthritis patients present an imbalance T-reg/Th17 ratio due to lower protectin DX levels than healthy donors. By inhibiting the NLRP3 pathway via miR-20a, Protectin DX restores this imbalance in the T-reg/Th17 ratio [136]. In models of lupus, NLRP3 promotes the differentiation of Th17 cells [137] and avoids T-reg differentiation through interaction with karyopherin subunit-α2 [138], leading to an imbalance T-reg/Th17 ratio.

### 4.2. Impact of NLRP3 in Immunosenescence and Adoptive Cellular Immunotherapy in Cancer

The NLRP3 inflammasome complex can accelerate immunosenescence, a mechanism mediated through lipids [139]. Thus, in obesity, NLRP3, through lipotoxic signals, such as free cholesterol and ceramides [140], causes an accelerated age-related thymic involution, leading to decreased T cell diversity with reduced naïve T cells and increased effector-memory T cells [141]. Moreover, aging causes an expansion of resident non-senescent aged adipose B cells that impairs tissue metabolism and promotes visceral adiposity in the elderly, a process dependent on NLRP3 [142]. Of interest, lipids [143] and pyroptosis [52] involvement have also been observed in the anti-tumor activity of NK cells against MM cells. This effect seems specific for only some tumor cells, as these events are not observed in the classic NK target, K562 cells [52,143]. On the other side, NLRP3 also has a beneficial impact on the adaptive immune response. Thus, CD8 T cells activate the NLRP3 in antigen-presenting cells (APCs) that promote IL1β maturation and contribute to the induction of antigen-specific anti-tumor immunity amplifying the CD8 effector functions [144].

The impact of NLRP3 in the development of immunosenescence and the immune response should be considered in adoptive cellular immunotherapy strategies to treat cancer patients. Indeed, adoptive cellular immunotherapy with CAR-T cells requires an in vitro activation and expansion, a process that changes their properties [50,145]. CAR-modified T cells activate pyropotosis when they encounter tumor cells [53]. Activation of pyroptosis by CAR-T cells when they face tumor cells leads to the release of factors that activate caspase 1 for GSDMD cleavage in macrophages [53], which results in massive amounts of cytokines, including IL6 and IL1β. IL6 and IL1β will trigger cytokine release syndrome and neurotoxicity, some of the main inflammatory complications after CAR-T cell therapy [146,147,148]. In addition, pyroptosis activation in tumor cells by CAR-T cells leads to the release of DAMPs, such as HMGB1, that also trigger macrophages to release IL1β and IL6 [53]. These findings should be considered when treating cancer patients to avoid the detrimental effects of this inflammation.

### 4.3. Impact of Inflammatory Granzymes Released by Immune Cells on Inflammaging and the Immune Response

Gzm are classically known as the mediators of the granule-dependent pathway killing of NK cells. However, they are also released by other immune cells. There are five different types of Gzm (A, B, H, K and M) in humans, and each activates different cell death pathways. Of interest, these molecules can trigger severe inflammatory reactions that can lead to autoimmunity and sepsis [56].

GzmK is released by CD56^bright^ NK cells [56] and by T cells. Specifically, GzmK-expressing CD8 T cells have been defined as a novel cellular hallmark of aging. These GzmK-expressing CD8 T cells are exhausted, express markers of tissue homing, and enhance the inflammatory functions of non-immune cells. In mice, GzmK CD8 T cells develop under an aged environment, where it is suggested that GzmK may increase inflammaging by exacerbating the SASP in fibroblasts [149]. Macrophages also express GzmK that by augmenting inflammation and impeding epithelialization influences wound healing [150].

GzmA also increases with aging, whereby being released by platelets controls the synthesis of increased IL8 and MCP-1 by monocytes [151]. In pathological conditions, elevated GzmA is observed in the serum of patients with peritoneal sepsis being involved in the development of the pathology. Specifically, GzmA, mainly expressed by NK cells, acts as a proinflammatory mediator in macrophages inducing TLR4-dependent expression of IL6 and TNFα [152]. GzmA also correlates strongly with inflammation and colorectal cancer. Thus, extracellular GzmA causes macrophages to release IL6 activating STAT3 in cells; and inhibiting extracellular GzmA attenuates gut inflammation, preventing colorectal cancer development [153]. However, on the other side, GzmA released by NK cells, T cells and CAR-T cells mediates anti-tumor activity in different gasdermin B-positive murine models through pyroptosis [55].

GzmM is expressed in NK cells, NKT cells, γδ T cells and 20–30% of the CD8 T cells [154]. GzmM inhibits the development of immunosenescence and inflammaging which are related to cytomegalovirus (CMV) infection [155,156]. On the other side, GzmM is released in the context of severe inflammation. Thus, in mice models of sepsis, NK cell-derived GzmM augments the inflammatory cascade downstream of TLR4, leading to IL1α, IL1β, TNFα and IFNγ secretion, which results in lethal endotoxicosis [157]. In models of endotoxemia, stimulation of whole blood with *Escherichia coli BL21*, *Pseudomonas aeruginosa* and *Neisseria meningitis* induced release of GzmM [158]. One study in patients with IBD showed that only patients with ulcerative colitis display high levels of GzmM exclusively in the inflamed distal part of the colon. To find out the role of GzmM, the authors designed models of experimentally induced-IBD with depletion of GzmM. The absence of GzmM increased parameters associated with severe intestinal histopathology and levels of inflammatory indicators, suggesting the protective role of GzmM at early stages of inflammation in IBD [159].

To summarize, different types of Gzm are released by immune cells after encountering pathogens or tumor cells to counteract them. However, at the same time, they will impact differently in the development of inflammaging and inflammatory diseases. Moreover, some of them present opposing roles in different contexts. Of interest, some of them are beginning to be studied in adoptive cellular immunotherapy that will bring with relevant information regarding their specific roles.

## 5. Association of Intrinsic and External Triggers of Inflammation with Cancer

As previously described, different studies have found that chronic inflammation is an essential trigger for cancer development [35,36]. Indeed, these associations are confirmed in meta-analysis studies. Specifically, very recently, a systematic review and meta-analysis were performed to find associations of inflammatory blood indicators with cancer incidence. A total of 103 studies were considered for the final analysis. Only longitudinal observational prospective or retrospective studies including cohort, nested-case control, case-control and nested-case cohort were contemplated. Colorectal cancer accumulated the highest number of studies describing a relationship between inflammation and cancer. This incidence was followed by breast cancer, lung cancer and prostate cancer. The most significant association of inflammatory parameters with cancer risk were detected for CRP (39.8%), fibrinogen (24%), IL6 (25%) and TNFα (24%) [160].

This association of chronic inflammation with cancer development is well exemplified in idiopathic inflammatory myopathies (IIMs). IMM is a chronic multisystem autoimmune condition that may cause muscle inflammation (myositis), skin manifestations and interstitial lung disease. Adult-onset IIMs associate with an increased risk of cancer. Indeed, around one in four patients are diagnosed with different types of cancer within three years before or after IIM onset [161]. In a cohort of IMM patients where the total mortality was 27%, it was identified that IIM-related causes of death were frequent (64%) and included cancer. The subtype IMM of dermatomyositis (DMM) was identified as an independent mortality risk factor. Specifically, cancer risk was increased in DMM and polymyositis but not in sporadic inclusion body myositis. Moreover, ovarian cancer was more prevalent in DMM than in the general population [161].

A meta-analysis of 69 different studies was performed to identify parameters associated with cancer risk in the IIMs and placed the transcription intermediary factor 1γ (ΤΙF1γ) protein [162]. ΤΙF1γ is known as a tumor suppressor. Of interest, auto-antibodies to ΤΙF1γ have a strong association with cancers associated with DMM. A clinical study in 160 DMM patients found that 26% of patients had cancer, being this proportion higher in the anti-TIF1γ-positive patients. Moreover, anti-TIF1γ-positive DM patients had more advanced cancers than anti-TIF1γ-negative DM patients. In addition, anti-TIF1γ-positive DM patients developed cancers closer to the time of the diagnosis of DMM than anti-TIF1γ-negative DM patients [163].These results suggest anti-TIF-γ auto-antibodies as a potential tumor auto-antigen and should alert the doctor to the possibility of underlying cancer.

**Figure 1:**
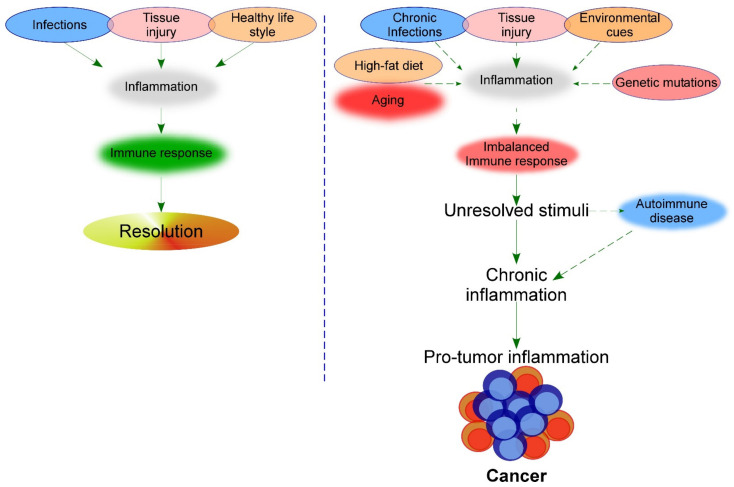
Intrinsic and external triggers that impact the immune response. Panel on the left shows insults that activate the immune response in a healthy life. Panel on the right shows different triggers that occur with aging that cause an inadequate immune response.

These studies exemplify the association of chronic inflammation with cancer development. However, even though the immune system response has an important role, as previously explained, additional triggers can accelerate the development of cancer (Figure 1). Some of these triggers are described in this section.

### 5.1. Diet

Diet has a relevant impact promoting an accelerated inflammation that leads to cancer, metabolic and neurodegenerative diseases. Dietary components can modulate glucose, insulin levels and other mediators that activate NF-κB to trigger inflammation through standard pathway master switches [164]. Thus, in breast cancer, a meta-analysis of seven observational studies has observed an association of a high “dietary inflammatory index” with a 25% of increased incidence of breast cancer [165]. Another meta-analysis study in urological cancers demonstrated that a high dietary inflammatory index was associated with higher cancer risk in prostate cancer, kidney cancer and bladder cancer. However, no associations were found for urothelial cell carcinoma [166].

Moreover, in obesity, inflamed adipocytes create a proinflammatory microenvironment with infiltrating immune cells that foster tumor progression through proinflammatory mediators, such as IL6, IL8 and IL1β. Of interest, biomarkers of adipose tissue inflammation can help to identify high-risk populations. Moreover, as adipose inflammation is a reversible process, novel therapeutic targets could be designed to break the obesity-cancer link [167].

### 5.2. Air Pollution

In vitro models showed that air pollution induces proinflammatory cytokines in human lung epithelial cells, particularly IL6 and IL8 [168]. A systematic review and meta-analysis study evidenced that long-term exposure to air pollutants increases the risk of cancer mortality. Specifically, for 10 μg/m^3^ per increase of particulate matter (PM)2.5, PM10 and NO_2_, overall cancer mortality risk estimates were 1.17, 1.09 and 1.06, respectively. Moreover, PM2.5 compromised lung cancer mortality, and non-lung cancer mortality, including liver cancer, colorectal cancer, bladder cancer and kidney cancer, while PM10 had dangerous effects on mortality from lung cancer, pancreas cancer and larynx cancer [169].

### 5.3. Genomic Instability

Genomic instability has also been defined as a trigger of aging [4] and cancer [38]. In this regard, in myelodysplastic syndromes (MDS), MDS-associated spliceosome mutations promote a proinflammatory state. This proinflammatory state is characteristic of MDS-derived myeloid cells through IL6 mRNA expression in an NF-κB-dependent manner [170]. Consistent with that finding, mutations in myeloid neoplasms in the splicing factors SF3B1 and SRSF2 enhance NF-κB activity [171] and lead to the activation of NLRP3 acting as a driver for MDS [172]. In agreement with this, the common mutant p53 isoform is frequently identified in human cancers. This mutant prolongs NF-κB activation, causes severe chronic inflammation and promotes inflammation-associated colon cancer [173].

## 6. Preclinical Studies That Associate Inflammation with the Development of Different Typesof Cancer

A variety of preclinical studies have demonstrated the association of chronic inflammation with different types of cancer in murine models. These models should be similar to humans in their clinical and histological features, share similar inflammatory or carcinogenic profiles, and be responsive to the standard treatment used in patients. For gastrointestinal cancers, different models have been reviewed and proposed [174]. In these models, gastrointestinal inflammation is induced orally with alcohol or other toxic agents, by injections of ulcer-inducing agents, parenteral or oral administration of carcinogens, or by modification of the genetic background of the animals. For instance, knock-out mice for IL10 develop spontaneous enterocolitis and cecal inflammation emulating human IBD [174].

Animal models have demonstrated that exposure to tobacco smoke promotes tumor development in both carcinogen-treated mice and transgenic mice in lung cancer. Smoking-associated inflammation and activation of NF-κB have been linked to the development of lung cancer [175]. Other reviews have proposed models where the role of peritumoral and intratumoral macrophages is considered; and where IL10, glucocorticoids, prostacyclin, nitric oxide and surfactant apoprotein D levels are proposed biomarkers to monitor tumor growth rate [176].

Of interest, a model of aged-mice deficient for granulocyte-macrophage colony stimulating factor (GM-CSF) develop a systemic lupus erythematosus-like disorder. This model, added to a deficiency of IFNγ causes diverse hematologic and solid neoplasms. Moreover, antimicrobial therapy prevents tumor development, demonstrating the interplay of infectious agents, regulation of immune homeostasis and cancer susceptibility [177]. Another murine model showed that loss of IFNγ, GM-CSF and IL3 induces chronic pulmonary inflammation and lung tumors with spontaneous activation of MAPK and STAT3 signaling, in addition to a critical dependence on NF-κB signaling. Moreover, IL6 promotes lung tumor growth, further implicating oncogenic inflammation in the development of tumors [178].

Additional murine models also showed that cigarette smoking increased ulcerative colitis-associated colonic adenoma formation [179]. Moreover, knock-out mice for Rag2 lack functional lymphocytes. In these models, lack of functional lymphocytes added to *Helicobacter hepaticus* infection induces chronic colitis and the development of colon cancer [180]. Hepatocellular carcinoma (HCC) is associated with pathogen infection-induced chronic inflammation. Murine models showed that the tumor suppressor gene STK4 regulates TLR3/4/9-mediated inflammatory responses in macrophages, protecting against chronic inflammation-associated HCC. These results suggested STK4 as a biomarker to target inflammation-induced HCC [181].

## 7. Summary

In conclusion, inflammaging is a relevant event occurring with aging that impacts the health of the elderly. Various intrinsic and extrinsic factors involved create a proinflammatory environment that influences the immune response’s proper function. Consequently, an uncontrolled immune response will increase the incidence of autoimmune diseases and subsequently develop cancer. A small number of clinical trials currently ongoing, and a variety of preclinical and observational pieces of evidence seem to confirm this association between the inflamed state of aging and cancer. Aging causes an intrinsic and natural defect in the thymus and the BM that will degenerate our immune cells. Of interest, additional triggers, which can be avoided, will accelerate this process. Essential balances between the different immune cells subsets must be maintained to prevent excessive immune responses with concomitant inflammation or inadequate immune responses against microbial or malignant transformed cells. Of interest, adoptive cellular immunotherapy is a continuously growing option of treatment for cancer patients. This therapy administers immune cells modified and in vitro expanded that significantly alter their properties and proinflammatory profile. Therefore, monitoring changes in these cell populations during treatment will provide relevant information for cancer treatment. In addition, studies regarding the immune response in the different fields of autoimmunity and cancer should be considered together to have a deeper understanding of the immune response.

## Figures and Tables

**Table 1:** Side effects of inflammaging.

Age-Related Diseases	Mediators	References
Atherosclerosis	Secretion of IL1β, IL18 and IL6 among others	[7,40]
Cardiovascular diseases	CRP and IL6 in blood	[7]
Frailty, Sarcopenia	Inflammatory markers in blood, IL6	[41]
Decline of innate and adaptive immune system	Immunosenescence	[8,9]
Type 2 diabetes	Secretion of IL1β among others	[34,42,43]
Cancer	CRP, IL6, immunosenescence	[7,25,26,35,36,44,45]
Osteoporosis, bone remodeling	IL1, IL6, TNFα	[46]
Neurodegenerative disease	Immune cells infiltration	[33]

## Data Availability

Not applicable.
